# Robotic-assisted excision of left para-aortic paraganglioma: a novel approach

**DOI:** 10.1093/jscr/rjae842

**Published:** 2025-01-21

**Authors:** Jack Kang Tan, Jason Ramsingh

**Affiliations:** Faculty of Medicine, Universiti Malaya, 50603, Kuala Lumpur, Malaysia; Department of Endocrine Surgery, Royal Victoria Infirmary, Newcastle Upon Tyne NE1 4LP, United Kingdom

**Keywords:** robotic, paraganglioma, daVinci, extra-adrenal, pheochromocytoma, retroperitoneal

## Abstract

Paragangliomas, a type of extra-adrenal tumour, albeit rare, are dangerous due to their high metastatic potential and risk of hypertensive crisis from massive catecholamine release. It typically presents with sympathetic overdrive symptoms such as diaphoresis, headache, and palpitation, accompanied by substantially high plasma metanephrines level and mass on contrasted computed tomography abdomen and pelvis, whilst some are found incidentally. In this report, we discuss a case of an extra-adrenal lesion located near susceptible major structures with extensive vascularisation, in a patient with near-death experience. Complete excision of the pulsatile mass with minimal bleeding and no complications, was made possible utilizing the da Vinci Robotic System. Robotic surgery, being a part of a multidisciplinary approach, leads to better patient outcomes and shorter hospitalisations. Moreover, it offers enhanced dexterity and improved depth perception compared to conventional methods. However, further studies are needed to validate its application in standard practice.

## Introduction

Despite the growing popularity of robotic surgery, there have been limited reports on paraganglioma excisions. Paragangliomas, tumours that derive from extra-adrenal chromaffin tissue, are commonly found at the organ of Zuckerkandl, located between the aortic bifurcation and inferior mesenteric artery [1–3]. Although having a low combined annual incidence of 0.8 per 100 000 person-years, and a prevalence of 6 per 1 million persons [4], it causes significant cardiovascular mortality and morbidity during hypertensive crisis from excess catecholamine release. According to the 8th edition of the American Joint Committee on Cancer on tumour, node, metastasis staging, all extra-adrenal paragangliomas are classified as T2, stage 2 tumours due to high metastatic potential [5, 6]. Hence, surgical resection remains the gold standard in treating paragangliomas to address secondary hypertension, improve glycaemic levels, and mitigate the risk of metastasis [1, 4]. This paper aims to document a case involving robotic extirpation for a pulsatile mass located at the organ of Zuckerkandl.

## Case report

**Figure 1:**
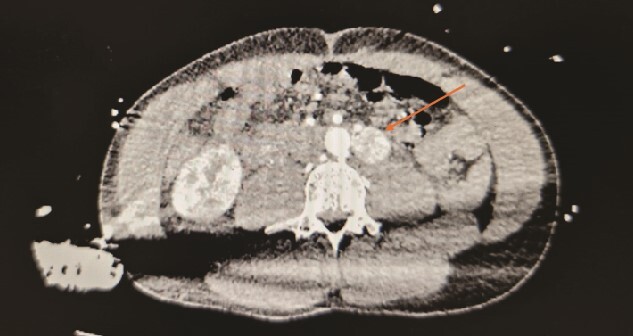
Contrasted CT in arterial phase, in the axial plane, showed a contrast enhanced, hyperdense, heterogenous lesion located over the left para-aortic region, measuring ⁓2 × 2 × 3 cm with no septations or peripheral rim enhancement.

**Figure 2:**
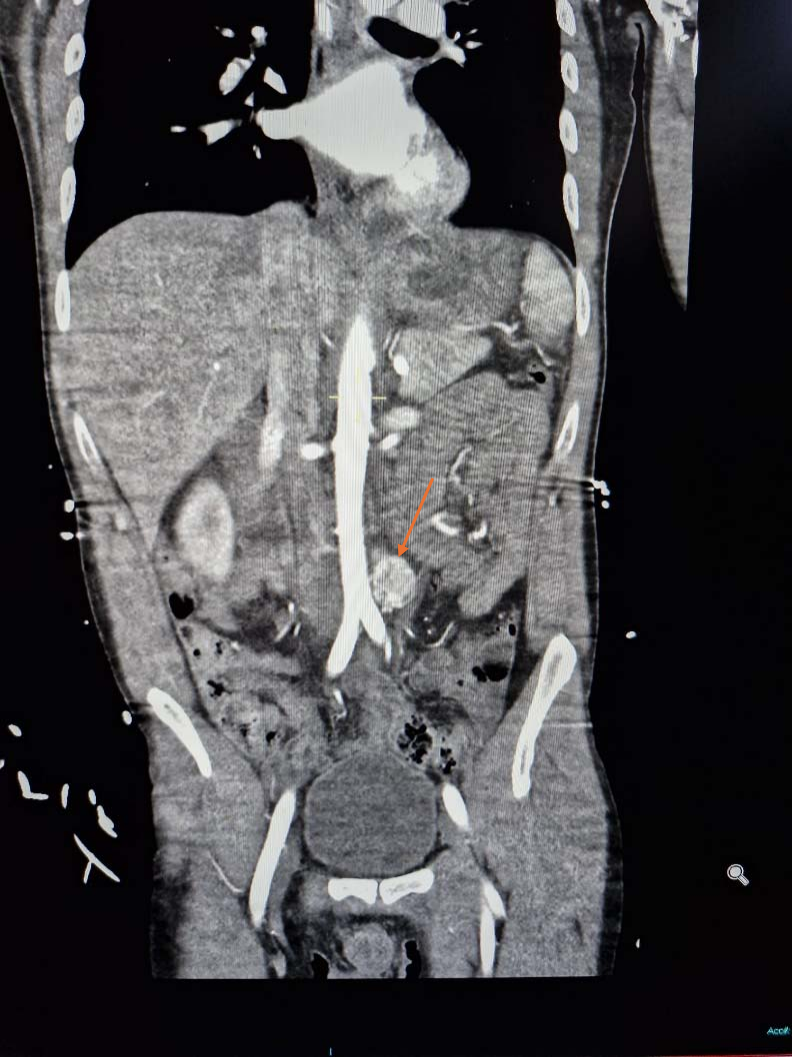
Contrasted CT of the abdomen and pelvis in arterial phase, coronal plane, revealed a similar mass, contrast enhanced heterogenous lesion with well demarcated borders, measuring ⁓2 × 2 × 3 cm, situated at the left para-aortic region.

A 30-year-old urology registrar presented to the emergency department complaining of headache, nausea and abdominal pain, and was found to be hypertensive. He became very agitated whilst on the ward and was intubated. A computed tomography (CT) of the brain was normal, but subsequent CT of his abdomen and pelvis revealed a 2 × 2 × 3 cm hypervascular mass adjacent to his aorta just above the bifurcation. Initially suspected to be an aneurysm, it was later identified as a paraganglioma (Figs 1 and 2). He was then referred to the endocrine surgery team.

The team advised on starting Doxazosin for blood pressure stabilization, after which he was extubated and discharged. His free plasma normetanephrine was markedly raised at 3600 pmol/L (0–510 pmol/L), whilst other parameters were normal.

Given the risk of hypertensive crisis causing stroke or sudden death, urgent tumour removal was advised. His consent was obtained after the surgical risks including bleeding, infection, damage to surrounding structures and perioperative risk of stroke, myocardial infarction and death was explained. He was also referred to a geneticist for genetic test counselling due to a family history of cancer.

Preoperative alpha receptor blockade with 10 mg Phenoxybenzamine was given three times a day for 4 weeks to optimize blood pressure.

**Figure 3:**
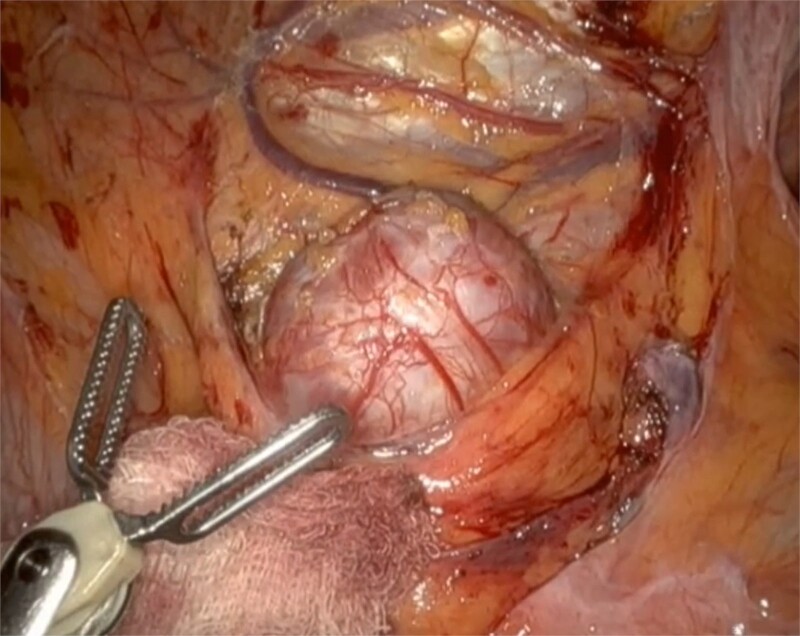
Well circumscribed mass with extensive vascularisation is located and isolated, with no surrounding tissue necrosis or invasion observed. Real time video footage revealed a pulsatile mass.

**Figure 4:**
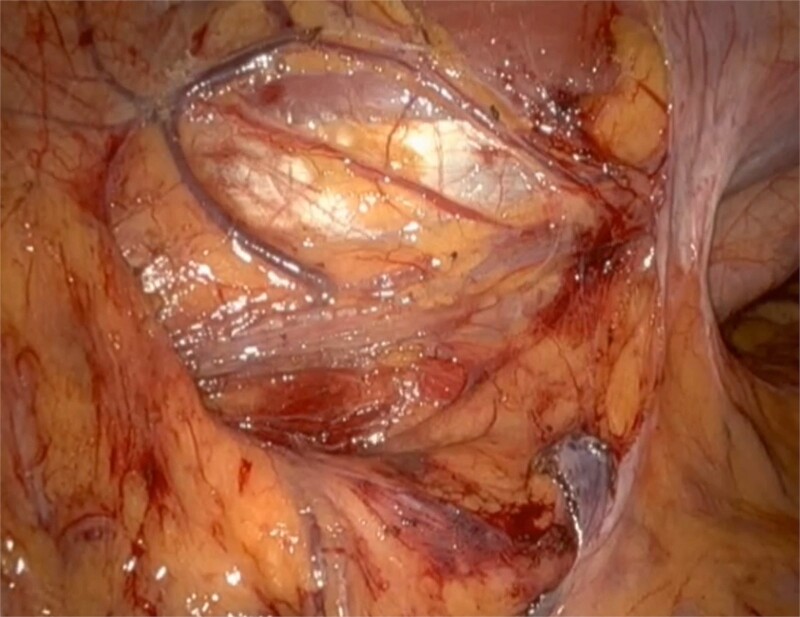
Post tumour removal showed clean base with no active bleeding. No evidence of tissue invasion into peripheral structures.

He underwent a robotic excision of his paraganglioma in a right lateral position, using a transperitoneal approach with three robotic ports and one assistant port in the midline. The left colon was mobilized medially, exposing the tumour. After identifying the ureter and gonads, the tumour was dissected freely using a vessel sealer, before removing it in a specimen bag. Operation was uneventful (Figs 3 and 4).

Histopathological lab reported a 35 mm sympathetic paraganglioma completely excised with clear margins, supported by presence of positive staining for synaptophysin, chromogranin, and S100 seen in sustentacular cells. Postoperative follow-up confirmed normal biochemical parameters with no complications.

## Discussion

Chromaffin cell tumours usually stem from adrenal medullas, with only 15% occurring extra-adrenally as paragangliomas [7]. Functional paragangliomas display sympathetic overdrive symptoms such as diaphoresis, headache, palpitations, and hypertension [3]. Free plasma metanephrines test is preferred for screening due to its high diagnostic accuracy and relatively simple process [1, 8].

CT scans of the abdomen and pelvis remain the preferred radiological method in diagnosing both active and silent paragangliomas, demonstrating 90% sensitivity and 93% specificity in identifying extra-adrenal tumours. These tumours typically appear as contrast-enhanced lesions with central hypoattenuation [8–10].

Preoperative administration of alpha-adrenergic receptor antagonist, followed by beta-adrenergic receptor to prevent unopposed stimulation of alpha-adrenergic receptor causing hypertensive crisis [8], is recommended for all patients to reduce intra-operative BP fluctuations, prevent cardiovascular complications, and lower overall mortality rate [2, 11].

Next, for definitive management, although some advocate open resection for paragangliomas due to its narrow site, malignant potential, presence of synchronous multifocal tumour, and need for contiguous organ resection, others suggested minimally invasive resection with laparoscopy or robotic arms to be the standard of care for small paragangliomas with clear resection margins [1, 8].

Laparoscopic retroperitoneal paraganglioma excision incurs difficulties due to technical limitations including restricted instruments’ range of motion, exacerbated by deep tumour location and its proximity to important structures [12]. Well equipped with 7 degrees of freedom, clear visual feedback in three dimensions, and stable robotic arms [2, 12], robotic surgery provides better dexterity allowing easy suturing and knotting, improved depth perception and spatial awareness, plus eliminates unstable instrument handling due to surgeons' fatigue with better ergonomics, enabling meticulous dissection, tedious haemostasis, and minimal tumour manipulation [3, 12, 13]. In addition to the United States Food and Drug Administration's approval [14], various studies on robotic excision of retroperitoneal tumours, reported positive outcomes including shorter postoperative hospital stay, lesser estimated blood loss, and lower conversion to open surgery, with similar morbidity and mortality rates, when compared to laparoscopic approach [3, 11, 12]. Interestingly, Naranjo *et al*. [11] reported similar treatment costs between laparoscopic and robotic surgery, factoring in the higher expenses of extended hospitalization, compared to more expensive robotic equipments. On the other hand, absence of tactile feedback and increased operative time may deter the usage of robotic surgery, which may be overcome with more training and experience [2, 11, 13].

Postoperative monitoring is imperative due to risk of hypotension from sudden drop in catecholamine levels and potential hypoglycaemia caused by hyperinsulinemia stimulated by chronic catecholamines excess [15]. Regular biochemical testing for catecholamine levels after surgery is essential for monitoring recurrence or metastasis [8].

Formal diagnosis of paragangliomas is confirmed through histopathology, with characteristic sustentacular cells stained positively for S100 and SOX10 [5]. Preoperative biopsies are avoided to minimize risks of inflammation and catecholamine leakage.

As paragangliomas are commonly associated with hereditary cancer syndromes, genetic testing shall be routinely offered [8].

In conclusion, paraganglioma extirpation is unique and intricate due to its hormonal properties, high metastatic potential, extensive vascularisation, and difficult location. Robotic surgery presents as a better alternative to conventional methods, by offering improved dexterity, better ergonomics, and clearer three-dimensional view whilst eliminating wobbly instrument usage. However, further studies on robotic paraganglioma excision are needed to confirm the safety and benefits of using robotic surgery in complex cases.
